# Intuition and Insight: Two Processes That Build on Each Other or Fundamentally Differ?

**DOI:** 10.3389/fpsyg.2016.01395

**Published:** 2016-09-13

**Authors:** Thea Zander, Michael Öllinger, Kirsten G. Volz

**Affiliations:** ^1^Department of Psychology, University of BaselBasel, Switzerland; ^2^Parmenides FoundationMunich, Germany; ^3^Department Psychology, Ludwig-Maximilians-Universität MünchenMunich, Germany; ^4^Werner Reichardt Centre for Integrative Neuroscience, University of TübingenTübingen, Germany

**Keywords:** intuitive decision making, insight problem solving, continuity, discontinuity, non-analytical solution processes

## Abstract

Intuition and insight are intriguing phenomena of non-analytical mental functioning: whereas intuition denotes ideas that have been reached by sensing the solution without any explicit representation of it, insight has been understood as the sudden and unexpected apprehension of the solution by recombining the single elements of a problem. By face validity, the two processes appear similar; according to a lay perspective, it is assumed that intuition precedes insight. Yet, predominant scientific conceptualizations of intuition and insight consider the two processes to differ with regard to their (dis-)continuous unfolding. That is, intuition has been understood as an experience-based and gradual process, whereas insight is regarded as a genuinely discontinuous phenomenon. Unfortunately, both processes have been investigated differently and without much reference to each other. In this contribution, we therefore set out to fill this lacuna by examining the conceptualizations of the assumed underlying cognitive processes of both phenomena, and by also referring to the research traditions and paradigms of the respective field. Based on early work put forward by Bowers et al. (1990, 1995), we referred to semantic coherence tasks consisting of convergent word triads (i.e., the solution has the same meaning to all three clue words) and/or divergent word triads (i.e., the solution means something different with respect to each clue word) as an excellent kind of paradigm that may be used in the future to disentangle intuition and insight experimentally. By scrutinizing the underlying mechanisms of intuition and insight, with this theoretical contribution, we hope to launch lacking but needed experimental studies and to initiate scientific cooperation between the research fields of intuition and insight that are currently still separated from each other.

## Introduction

There are situations, in which decision makers arrive at an idea or a decision not by analytically inferring the solution but by either sensing the correct solution without being able to give reasons for it, or by realizing the solution all of a sudden without being able to report on the solution process. Roughly, the former phenomenon has been called intuition, the latter insight. Both have fascinated the public as well as the scientific audience.

Here are two historical cases that illustrate the two phenomena (Gladwell, 2005; Mclean, as cited in Klein and Jarosz, 2011): The first is known as the Getty kouros and happened to the J. Paul Getty Museum in Los Angeles at the end of the 20th century. The museum was offered to add an over-life-sized statue in form of a kouros – allegedly from Ancient Greece, and thus several millions worth – to its art collection. Before the contract could be concluded, several experts set out to assure the authenticity of the statue and its origin thereby using a substantial number of high-tech methods for their analyses. After a year of thorough inspection, the experts reached the conclusion that the statue was authentic. At the same time, the former curator of the Metropolitan Museum of Art in New York, by chance, cast a glance at the artwork and spontaneously raised doubts regarding its authenticity. Thereupon, other men of renown who were asked for their spontaneous assessment of the kouros, also reported that they *felt that something was wrong* with it – without being able to tell the reason for this impression (cf. Gladwell, 2005). Interestingly, up to now, it could not be entirely cleared whether the statue stems from Ancient Greece or whether it is a modern forgery. Yet, the curator – instantaneously “feeling” that something was wrong and acting upon this impression although not being able to name a specific reason – is a paramount example of what it means to have an intuition being strong enough to act accordingly.

For an example of a sudden insight into the solution of a complex problem, consider Wagner Dodge, a smokejumper who survived the Mann Gulch Fire in August 1949 (Mclean, as cited in Klein and Jarosz, 2011). On a very hot day, a fire broke out in Mann Gulch, a canyon near Helena in Montana. Sixteen smokejumpers were flown close to the fire in order to extinguish it. After they had parachuted out of the aircraft, they realized that the fire was much worse than expected: They faced an uncontrollable blaze. The biggest problem was that they were in the danger of being entrapped by the fire. They could not escape and thus their lives were immediately threatened. For a moment they were desperately helpless and bustled around without a plan. They faced an *impasse*: well-known routines would not bring them forward and they might be caught in a *mental set*, that is, the tendency to try to solve a problem based on previous successful solution attempts to similar kinds of problems that are inefficient or cannot be transferred to the problem at hand (see Luchins and Luchins, 1959, as well as Öllinger et al., 2008). After a while, all at once, Wagner Dodge had the sudden idea to ignite an “escape fire” ahead of the group (i.e., he had a sudden *aha-experience*). Although he had never heard of such a possibility, he *abruptly realized* that when he could quickly stub an area of vegetation, the blaze would have no basis to continue when arriving at the cinder. He put his idea into action, ignited an additional fire and stepped into the middle of the newly burnt area. This way, he could save his life; the other smokejumpers who did not trust him lost their lives in the fire. Today, escape fires belong to the standard practice of fire services in the wild (Mclean, as cited in Klein and Jarosz, 2011).

Based on these examples, both phenomena – intuition and insight – may be conceived of as *non-analytical thought processes* that result in certain behavior that is not based on an exclusively deliberate and stepwise search for a solution. Non-analytical thought means a thought process in which no deliberate deduction takes place: individuals are not engaged in the consecutive testing of the obvious and/or typical routes to solution that define deliberate analysis. Instead, intuitions are characterized by the decision maker feeling out the solution without an available, tangible explanation for it; insights are characterized by the fact that the solution suddenly and unexpectedly pops into the mind of the decision maker or problem solver being instantaneously self-evident. Despite these apparent similarities of the two phenomena, intuition and insight have been conceptualized rather differently in the scientific literature up to now with regard to the underlying cognitive mechanisms as well as to the experimental designs routinely being used to gain empirical evidence. The aim of our contribution is therefore to scrutinize the similarities and differences of the cognitive mechanisms underlying intuition and insight by drawing on and extending early ideas by Bowers et al. (1990, 1995). The gripping question is whether intuition and insight are two qualitatively distinct phenomena, appearing similar only by face validity, or whether they are indeed similar/related and may only unfold on different levels of processing. To address this question, we draw on the latest contributions in the field and include recent research findings that have not been available in Bowers et al. (1990, 1995) time.

First, we will give an overview of predominant definitions of intuition and insight from a cognitive-psychological perspective. Second, we will elaborate on the underlying cognitive processes of both phenomena, thereby aiming to pin down similarities and differences. Both, similarities and differences will be addressed against the background of the research history of intuition and insight as well as in light of predominant, experimental paradigms that have been used to investigate the two phenomena. The paper ends by outlining open questions and highlighting future directions in scientific research that may progress our understanding of the underlying cognitive processes of intuition and insight (as well as on their relatedness).

## Defining Intuition and Insight

### Theoretical Characterization of Intuition

Although most people “intuitively” know what an intuition is, the scientific community is split over its definition as well as its conceptualization. Despite disagreement about any definition, common ground is that intuition is an experienced-based process resulting in a spontaneous tendency toward a hunch or a hypothesis (Bowers et al., 1990; Volz and Zander, 2014). Taking all major definitions into consideration, it is possible to distil certain characteristics that prominent definitions of intuition have in common (Glöckner and Witteman, 2010; Volz and Zander, 2014).

Firstly, there is the aspect of *non-conscious processing*, which means that intuition occurs with very little awareness about the underlying cognitive processes so that people are mostly not able to report on these. Yet, intuitive processes can partly or completely be made conscious at some point in the entire judgmental process (e.g., Gigerenzer, 2008). In this regard, intuitive processing is not directly conscious or non-conscious, but can be viewed as reflecting cognitive processing on the *fringe of human consciousness* (Mangan, 1993, 2001, 2015; Norman, 2002, 2016; Price, 2002; Norman et al., 2006, 2010). Secondly, there is the aspect of *automaticity* or *uncontrollability*. Intuitive processing appears in the form of spontaneous and instantaneous ideas or hunches that cannot be intentionally controlled in the way that they cannot be neither intentionally evoked nor ignored (e.g., Topolinski and Strack, 2008). The unintentional nature of intuition implies that intuition comes along without attentional effort and thus intuitive processing has been described as fast and effortless (e.g., Hogarth, 2001). Thirdly, there is the aspect of *experientiality*. Intuitive processing is based on tacit knowledge that has been acquired without attention during a person’s life and is thus fueled by it (e.g., Bowers et al., 1990). In combination these aspects result in the subjective experience of “knowing without knowing why” as Claxton (1998, p. 217) put it. Lastly, there is the aspect of the *initiation of action*. The non-conscious, experience-based, and unintentional process finally results in a strong tendency toward a hunch, which serves as a go-signal that is strong enough to initiate action. As a result, people act in accordance with their intuitive impression or feeling (e.g., Gigerenzer, 2008). For a more detailed overview of the different aspects, consult Glöckner and Witteman (2010) or Volz and Zander (2014).

In line with these aspects, Gigerenzer (2008) has focused, inter alia, on the experiential basis of intuition and states that intuition may hardly be possible without pre-existing knowledge and experiences. To revert to the example of the Getty kouros, the interplay of the given (visible) information was dissonant for someone who had seen lots of antique statues before; a beginner to the field may have arrived at a completely different judgment. By intuitively apprehending the situation, the curator relied on specific long-term-memory content that had been primarily acquired by studying, analyzing, and reflecting about a great number of statues resulting in associative and unattended learning. Volz and Zander (2014) refer to this kind of memory content as *tacitly (in)formed cue-criterion relationships*. On this view, different environmental cues can have different predictive power with respect to the criterion at hand; the situational validity of the cues will moderate whether the cue is used outright. In the above example, the curator judged the grade of authenticity of the kouros (criterion) from the subjective impression that the statue’s outer appearance had on him (cue). By doing this, the curator could not only rely on the given information (i.e., the visible kouros), but had to non-consciously activate further relevant knowledge from memory, that is to activate associatively learned cue-criterion relationships. Thus, the mental representation constructed during intuitive processing goes beyond the existing, perceivable information. Consequently, the curator’s feeling of unease when having a look at the statue resulted from an incomplete cue-criterion relationship that was taken as diagnostic for the assessment of the statue’s authenticity.

In addition to the aspect of experientiality and the unconscious read-out of implicitly learned cue-criterion relationships, Gigerenzer (2008) describes intuition as *felt knowledge* that aids decision making not only in cases, in which the decision maker already has a huge amount of prior experiences with a particular situation, but also when time and cognitive capacity is limited. According to the author, shadowy situations – either caused by a blurry sensory input that is only hardly detectable, or by the temporary non-availability of necessary information about the individual decisional components, which does not allow for foreseeing all consequences of a decision – foster intuitive processing. Intuition then manifests itself in the use of certain heuristics that may form highly successful, cognitive shortcuts (Gigerenzer, 2008; Gigerenzer and Gaissmaier, 2011).

### Insight and Aha-Experience

In contrast to the above elaborations on intuition, the term insight has been used to refer to the *sudden and unexpected understanding* of a previously incomprehensible problem or concept. In this sense, Jung-Beeman et al. (2004, p. 506) explicate the nature of insight as “the recognition of new connections across existing knowledge.” Sometimes the solution to a difficult problem may suddenly pop out in the mind and the decision maker or problem solver may immediately recognize the complex nexuses, as formerly illustrated in the episode of the smokejumper Wagner Dodge. Problems seem to be processed and solved by re-grouping or re-combining (i.e., re-structuring) existing information in a new way so that *self-imposed constraints* can elegantly be relaxed (Duncker, 1935; Wertheimer, 1959; Ohlsson, 1992). Wagner Dodge had prior knowledge: For instance, he knew how fires most commonly can be extinguished and that fires need vegetation or some other foundation to burn on. Furthermore, he knew about terrestrial conditions, and most important, he knew that smoke and fire could kill him. The solution to the problem occurred when he non-consciously combined all pieces of knowledge with each other in a new way so as to circumvent the fire death.

Such insightful solutions are associated with a privileged storage in long-term memory. Likewise as single trial learning. Recent studies observed a memory advantage for items that were solved by insight compared with non-insight solutions (Danek et al., 2013) as well as compared with items that were not self-generated (Kizilirmak et al., 2015). So, it is very likely, that Wagner Dodge never forgot how to ignite escape fires in the wild.

Yet, it has to be emphasized that an exact definition of the term insight has proven to be difficult, not least because the term insight has been used in many different ways in problem-solving research. Another hindrance is that it is very difficult to empirically operationalize the psychological construct of insight (Knoblich and Öllinger, 2006), which is a similar problem as in research on intuition. Hitherto, researchers disagree whether there are certain necessary and/or sufficient conditions to determine whether an insight has occurred. For example, due to the absence of objective physiological markers indicating the occurrence of an insight, mainly reports in form of the *subjective aha-experience* have been used *ex post* to determine whether an insight has occurred during the solution process of a certain problem (e.g., Gick and Lockhardt, 1995; Bowden et al., 2005; Danek et al., 2013). Danek et al. (2013, p. 2) state that the aha-experience is “the clearest defining characteristic of insight problem solving.” Topolinski and Reber (2010) define the aha-experience as the sudden and unexpected understanding of the solution, which comes with ease and is accompanied by positive affect as well as confidence in the truth of the solution. Given scientific endeavors to (objectively) pin down whether an insight had occurred, it can be summarized that insight and aha-experience have been equated. However, to date, there is disagreement whether (a) every insight is accompanied by an aha-experience, and (b) aha-experiences can only accompany insights and do never occur for presented solutions (i.e., solutions that are not generated by the individual herself; cf. Klein and Jarosz, 2011; Kizilirmak et al., 2015).

In order to help clarifying the conceptual muddle on insight, Knoblich and Öllinger (2006) proposed a classification of insight on three dimensions: first, on a phenomenological dimension, insight is opposed to a systematic and stepwise solution approach. Instead, it can be described as the sudden, unintended, and unexpected appearance of a solution idea, which is accompanied by a strong emotional component – the subjective and involuntary aha-experience. Second, on a task dimension, the literature on insight distinguishes between predefined insight problems and non-insight problems, with insight problems requiring sudden solution ideas and non-insight problems requiring a rather incremental solution approach. In case such an insight problem is solved, it is inferred that it is very likely that an insight has taken place. For example, the nine-dot problem (Maier, 1930), the eight-coin problem (Ormerod et al., 2002), and the candle problem (Duncker, 1935) belong to such classical insight problems. However, a disadvantage of this distinction is that there are no unique criteria for an insight problem, and most of these problem could be solved with or without having an insight (Öllinger et al., 2014); the most proposed criteria refer back to the subjective experience of aha, which has led to a circular definition of insight and insight problems. To circumvent this disadvantage, Bowden et al. (2005) have suggested using a class of problems that can be solved either with insight or without insight. Last, on a process dimension, recent research is concerned with the underlying cognitive mechanisms of insight and how these are different from non-insight problem solving. The predominant assumption here is that the non-conscious cognitive process of a mental set shift enables a changed representation of the problem’s elements (Ohlsson, 1992, 2011), which in turn leads to a sudden insight into the solution. For instance, in the nine-dot problem, the sudden realization that moves beyond the virtual nine-dot square are possible may lead to the relaxation of the perceptually driven boundary constraints and thus to a representational change of the problem space, which in the following enable insightful solutions (for a detailed explanation of the three dimensions consult Knoblich and Öllinger, 2006)^1^.

## Different Research Traditions of Intuition and Insight

After having defined both cognitive phenomena, intuition and insight, it becomes obvious that both share a similarity in terms of persisting conceptual difficulties. Moreover, with regard to the subjective phenomenology they reveal a distinct picture: While intuition means to non-consciously understand environmental patterns and to act according with this first impression without being able to justify it (Bowers et al., 1990), insight problem solving deals with situations in which a solution pops into a person’s mind out of the blue (Durso et al., 1994). Yet, both processes can be viewed as non-analytical solution or thought processes, where no incremental search takes place. In the following, we will critically elaborate on the cognitive processes assumed to underlie intuition and insight. Starting point will be a few words on the research history of both, which allow to understand why both fields of research have developed independently over time.

### The Single- vs. Dual-System View on Intuition

Intuition research has been deeply integrated in research on judgment and decision making that investigates how humans decide between alternatives and judge situations (Plessner et al., 2008). Yet this took some time, in which intuition had been neglected due to its elusiveness (Betsch, 2008). Now researchers agree that “intuition need not to be “magical” – it can be defined and explained scientifically” (Sadler-Smith, 2008, p. 1). It has to be emphasized, though, that, historically, the concept of intuition has fallen between (at least) two stools: The *fast-and-frugal-heuristic* approach – which sees the concept in a positive light as it serves as the basis for heuristics and thus is a valid strategy successfully be used when time and cognitive capacity is limited in a fuzzy real world (Gigerenzer et al., 1999) –, and the *heuristics-and-biases* approach – which conceives of heuristics based on intuition as a source of erroneous and biased thinking that demonstrates human cognitive fallibility (Kahneman and Tversky, 1974). Both approaches have localized the concept of intuition completely differently within human thought processes and assign qualitatively different functions to it. Today, due to their continuing, fundamentally contradictory assumptions concerning human cognition, the fast-and-frugal-heuristic approach and the heuristics-and-biases approach pit themselves against each other. Conceptually, the key difference may be that Kahneman and Tversky (1974) and Kahneman (2011) advocate a dual-system view on human thinking (intuition vs. deliberation), whereas Kruglanski and Gigerenzer (2011) and Mega et al. (2015) favor a single system view of unified processes in thinking and reasoning. Additionally, it has to be emphasized that, since interest in intuition has mainly originated from the area of judgment and decision making, implications for intuition with respect to problem solving processes (and insight) are rather hard to derive from this kind of research. This may have complicated experimentally clarifying the relationship between intuition and insight.

### Intuition As Experienced-Based Perception of Coherence and As an Antecedent of Insight

To anticipate elaboration taking place later in this contribution, we mention a third approach in intuition research, which has developed independently from any dual- or single perspective and has its roots in the creativity and problem-solving literature (Mednick, 1962; Bowers et al., 1995; Dorfman et al., 1996). Intuition is here conceived as the experience-based perception or recognition of environmental meaning/coherence in terms of a sensitization toward the detection of hidden patterns whose structure cannot be immediately verbalized. For example, in the different versions of the semantic coherence task originally developed by Bowers et al. (1990), participants are asked to judge the semantic coherence of word triads and to name a forth word that may be the semantic link between the words, if it exists. Research found out that in these tasks participants are able to correctly categorize word triads as semantic coherent or incoherent – intriguingly even when they are not able to name the forth word, which is a paramount example of intuitive processing (e.g., Bowers et al., 1990; Bolte and Goschke, 2005). They rather feel the semantic link between the three words, but are not (yet) able to report on the reasons in terms of a solution concept that describes the semantic associations between the triad’s constituents. The concept of fringe consciousness (Mangan, 1993, 2001, 2015) may be helpful to further understand intuition as the preliminary perception of environmental coherence. Price and Norman (2008), referring to the concept of fringe consciousness, have explained that the stream of consciousness does not only include a *nucleus of consciously available information*, but also a *non-conscious fringe* that contains cognitive signals of temporarily unavailable, non-conscious information processing that is constantly going on in the background (as it accompanies cognition). These signals are continuously going on as *cognitive byproducts of cognitive processes*. Yet, they are only consciously experienced when attention is drawn to them (Reber et al., 2004). Regarding the semantic coherence task, the product of this non-conscious processing on the fringe (i.e., the subjectively experienced intuition) is consciously perceivable, but its antecedents, direct content, and underlying processing mechanisms are outside of awareness (see also Topolinski and Strack, 2009a).

On this view, intuitive responses have been understood as “intuitive antecedents of insight” (Bowers et al., 1995, p. 27). As far as we know, this has been the first (and only) conception that up to now has addressed a potential link between intuition and insight. Their early work allows deriving assumptions concerning the interaction of intuition and insight in more detail. Moreover, this conceptualization produced valuable empirical paradigms (e.g., semantic and visual coherence judgment tasks) that are particularly suited to investigate insight and its intuitive precursors. Therefore, we will elaborate on this conception later in this contribution when aiming to clarify the conceptual relationship between intuition and insight^2^.

### The Special-Process vs. Nothing-Special View on Insight

In contrast, research on insightful thinking has its roots in Gestalt psychology, which investigated the integration and ordering mechanisms of human perception and problem solving (e.g., Köhler, 1921; Duncker, 1945; Metzger, 1953). Similar to intuition research, the research on insight problem solving is also located between two different views: The *special-process view* – which posits that insight problem solving involves a unique cognitive process that is qualitatively different from the processes non-insight problem solving utilizes – and the *business-as-usual* or *nothing-special view* – which assumes that mainly the same cognitive processes are involved in insight and non-insight problem solving (Seifert et al., 1995). Despite these two views, scientists have been highly fascinated by the topic since its early description by the Gestalt psychologists. This great interest culminated in the seminal book “The nature of insight,” which mainly deals with the Gestalt psychologist’s view on insight problem solving (Sternberg and Davidson, 1995).

### Interim Summary I

In sum, both concepts, due to their elusiveness, had to fight for recognition as an established field of research. Nevertheless, regrettably, research on intuition and research on insight has developed mostly independently from each other. However, this is in sharp contrast to a lay perspective on the two phenomena, which would rather endorse the perspective that intuition and insight are inherently intertwined with intuition being an antecedent of insight (in terms of a slight previous impression on the fringe of consciousness). Yet, the two branches of research evolved from different research traditions using different scientific paradigms and, unfortunately, have referred to one another only marginally (i.e., for instance by Bowers et al., 1990). Therefore, we think it is now time to scrutinize the relationship between the two phenomena in greater depth. Based on Bowers et al. (1990, 1995) work, we will do this by elaborating on the cognitive similarities and differences of the two phenomena and by offering preliminary process ideas on their relationship.

## Differences in the Cognitive Processes Assumed to Underlie Intuition and Insight

### The Continuity Model of Intuition: Intuition As a Gradual Process

In the majority of conceptualizations, intuitive processing has been described within a continuity model locating intuition on one end of the continuum and insight on the other. A prominent example is the two-stage model put forward by Bowers et al. (1990). The authors determine intuition as the preliminary perception of coherence in the environment triggered by tacit knowledge that has been acquired unintentionally during a person’s life (i.e., the cue-criterion relationships that we addressed earlier in this contribution, see also Volz and Zander, 2014). While tacit, or implicit, knowledge is seen as the foundation on which intuitions are based (e.g., Lieberman, 2000), in our view, intuition must not be regarded solely as a phenomenon of or even be equated with implicit memory processing. As Volz and Zander (2014) clarify, there are several important differences between intuition and implicit memory concerning both the format in which information is stored in memory and the kind of signal that accompanies the respective cognitive process. The fact that implicit knowledge is seen only as one component of processing is similar to the field of implicit cognition in general. Here, implicit knowledge is assumed to be supplemented and/or completed by antecedent hunches of correct solution, the *subjectively experienced nearness to the solution* (Reber et al., 2007).

Based on Polanyi’s (1966) concept of tacit knowledge, Bowers (1984, p. 256) defined intuition as “sensitivity and responsiveness to information that is not consciously represented, but which nevertheless guides inquiry toward productive and sometimes profound insights.” According to the author, the cognitive processing from an intuitive hunch toward an explicit insight is gradual and proceeds in two stages. In the first stage, the *guiding or intuitive stage*, environmental cues trigger the activation of tacit knowledge associatively connected in semantic memory, which results in an implicit perception of coherence that (yet) cannot be explained verbally. This process is characterized by the automatic spread of activation proposed by Collins and Loftus (1975). In the second stage of intuition, *the integrative or insight stage*, information becomes consciously available, which is enabled via a gradual accumulation of the previously activated concepts. The previous, implicit activation becomes now explicitly represented, which may thus be also interpreted as a form of insight processing. Hence, in Bowers et al. (1990, 1995) conception, intuition precedes insight in the way that explicit representations are anticipated by the sensitization of environmental pattern or structure. Yet, besides the idea of a gradual, successive accumulation of activated concepts in associative memory, unfortunately, it has remained unclear which cognitive and/or physiological conditions foster the transition from sensed intuition to justified insight.

Bowers et al. (1990) approach is not only theoretically important it also carries paradigmatic weight. In order to empirically test their model’s assumptions, the authors developed several novel paradigms (verbal as well as perceptual ones), which today, after slight revisions, belong to the standard paradigms of intuition research (e.g., Bolte and Goschke, 2005; Volz and von Cramon, 2006; Topolinski and Strack, 2009b; Hicks et al., 2010; Remmers et al., 2014; Zander et al., 2015). One of them is the semantic coherence task mentioned above, consisting of word triads that can be either semantically coherent (e.g., SALT, DEEP, and FOAM) or incoherent (DREAM; BALL; BOOK). Semantic coherence is determined via a fourth word each word of the word triad’s constituents associatively hints at (e.g., SEA for the coherent triad). Participants are instructed to perform a *semantic coherence judgment*, that is, to indicate via button press whether a given triad is coherent or incoherent. Researchers found that people showed an above-chance discrimination between coherent and incoherent triads even when they are not able to name the forth word (e.g., Bowers et al., 1990; Bolte and Goschke, 2005). In other words, people were intuitively sensitized to the detection of coherence prior to its explicit recognition (i.e., before having an explicit insight into the underlying semantic structure). Using a similar task, which consists of up to 15 semantically target-related clue words (i.e., the Accumulated Clues Task), it could be observed that participants *continuously approached the explicit representation* of environmental patterns/meaning (Bowers et al., 1990; Reber et al., 2007), which could be recently also demonstrated on a neuronal level when using the semantic coherence task (Zander et al., 2015). These results are perfectly in line with Bowers et al. (1990) definition of intuition and the corresponding gradual two-stage model. As another important aspect concerning the link between intuition and insight, Bowers et al. (1990) suggested the concept of semantic convergence to differentiate between triads that are rather easily solved by non-consciously reading out the common association (i.e., convergent triads) and triads that require a reorganization of semantic associations (i.e., divergent triads; see also the section *Bridging the gap between the underlying processes of insight and intuition*, second part).

To put it in a nutshell, according to the continuity model, – as Bowers et al. (1990) defined and tested it by means of verbal and visual coherence tasks – intuition and insight (in terms of an explicit representation that can be verbalized) are inherently intertwined: intuition and insight build upon each other and the one can hardly occur without the other. That is, intuitive processing is the non-conscious precursor of insight and thus, intuition and insight build on each other evolving on different processing stages. Accordingly, intuition and insight are not considered qualitatively distinct or mutually exclusive. Instead a crosstalk between the two is possible and even required to some extent. Importantly, Bowers et al. (1995) noted, that a thought process that appears to be sudden on a phenomenological level (like an aha-experience) nevertheless could have continuous underlying processes that have led to the particular subjective experience. Thus, they do not exclude the existence of subjective aha-experiences accompanying the successful solution generation in their verbal tasks.

Along these lines, when investigating insights from a naturalistic perspective (i.e., in a field setting and not in controlled laboratory settings), Klein and Jarosz (2011) found out that a substantial number of insights occurred gradually and in an (non-conscious) evidence-accumulating fashion. Following the naturalistic-decision-making approach (Zsambok and Klein, 1997), the authors aimed at investigating the *natural occurrence of insights* by analyzing a collection of reported insight incidents (comprising a radical shift in understanding) having occurred in the different domains of everyday life of different occupation (e.g., invention, firefighting, management, and the like). The authors found out that (a) impasses did not occur in each insight case, (b) not every incident of an insight was accompanied by an aha-experience, and (c) an intuitive feeling of how near the solution might be occurred in many cases before the actual solution was reached. These results indicate that insights in a naturalistic setting may differ from insights synthetically induced by the class of pre-defined insight problems (e.g., eight-coin-problem, Ormerod et al., 2002) according to the degree with which the solution is derived gradually. Thus, in the naturalistic setting, a continuous solution approach (as advocated in intuition research) may be adoptable.

### The Discontinuity Model of Insight: Insight As the Result of a Mental Restructuring Process

Contrary to the idea of a gradual solution approach, there is the discontinuity model of problem solving: insight is strongly linked to cognitive processes that restructure mental problem representations in order to allow the generation of a solution to a complex problem. A prominent example of a discontinuity model is the *representational change theory* put forward by Ohlsson (1992, 2011) that combines the Gestalt psychological approach (characterized by a person being unable to report conscious solution strategies, cf. Duncker, 1945) and the information-processing view on problem solving (characterized by a conscious search through alternatives in a problem space, which is a controllable and reportable process, cf. Newell and Simon, 1972). According to the representational change theory, and in sharp contrast to the two-stage model developed by Bowers et al. (1990), prior knowledge and experiences are postulated to hamper (instead of promote) the generation of solutions since they easily turn into constraints (Knoblich et al., 1999). Based on this, Ohlsson (1992) introduced the idea that an impasse, that is a “blind lane” where one is caught in wrong solution attempts finding no expedient or problem solving attempts ceases, is the precondition for a representational change that results in an insight. According to the author, a *restructuring process* is required, during which self-imposed constraints of the problem representation change and the problem solver obtains a “fresh look” at the problem. Problem solvers may then be able to rearrange either the individual components or the general assumptions how to solve the problem. A putative mechanism assumed to drive such restructuring processes is the *relaxation of self-imposed constraints*. The representational change theory became very influential; there are several studies that have tested and could corroborate its assumptions (e.g., Knoblich et al., 2001; Kershaw and Ohlsson, 2004; Öllinger et al., 2006, 2013).

In an eye movement study, for example, participants were asked to transform an incorrect arithmetic statement, which is made up of Roman numbers made of matchsticks, into a correct one moving only one single matchstick. Interestingly, it could be observed that before the correct solution of difficult problems was generated, suddenly, solvers attended such problem elements of the equation (e.g., the operators) longer that they had hardly noticed before. This was taken as evidence that successful solvers overcame self-imposed constraints (Knoblich et al., 2001). Research on the underlying cognition of the representational change theory could also help in understanding the subjective aha-experience as a subjective marker of insight: a recent study conducted by Danek et al. (2016) provides first evidence that the self-reported rates of aha-experiences depend on the degree of constraint relaxation that is necessary to solve the given problem. The authors found that the more constraints had to be relaxed, the less aha-experiences were reported, which was interpreted such that the execution of several necessary solution steps (that are needed to gain a representational change) minimizes or even eliminates the experience of suddenness as a key attribute of subjective aha-experiences.

### Interim Summary II

To summarize, according to a discontinuity model, the cognitive processes of intuition and insight seem to be qualitatively distinct. No crosstalk between them is possible. Moreover, the first (intuitive) look on a problem resulting in a mental impasse biases the subsequent solution. To be more precise, the intuitive apprehension of a problem necessarily leads to an impasse and restructuring processes are needed so as to overcome the bias and to solve the problem. This can be demonstrated, for example, via the utilization of magic tricks in order to probe insight problem solving. To explicate, Danek et al. (2013) recently introduced a novel paradigm consisting of magic tricks to investigate the cognitive underpinnings of insight problem solving. When viewing these magic tricks, the intuitive viewing pattern, which the magician intentionally utilizes, will very likely prohibit the understanding of the trick, that is, to first impede the solution to the problem. The solution is only within reach when the intuitive apprehension of the magic-trick situation, that is the first and rapidly formed impression, can be overcome. Classical insight problems as for example the famous candle problem (Duncker, 1935) utilize the same rationale.

## Bridging the Gap between the Underlying Processes of Insight and Intuition

### Dual-System Models of Thinking and Reasoning

This discontinuity approach resembles the experimental procedure in typical judgment and decision-making studies conducted within the heuristics-and-biases framework (Kahneman, 2011). This framework draws on a class of psychological models that are very well known in social and cognitive psychology and are called dual-system or dual-process models (e.g., Evans and Frankish, 2009; Kahneman, 2011). These models assume two different modes of thinking, which Stanovich and West (2000) called System 1 (described as e.g., non-conscious, fast, associative, holistic, automatic, and emotional) and System 2 (described as e.g., conscious, slow, analytic, serial, controlled, and affect-free). In other words, according to dual-system models, judgments may be formed via two qualitatively distinct processes or systems – an intuitive one (System 1) or a deliberate one (System 2). The intuitive strategy, thereby, is thought to require some sort of a feeling that “tells” a person which option is the optimal one. Thus, affective feelings are here seen as a crucial component that is inherent to the entire decision process. In contrast, when thoroughly deliberating on the pros and cons of multiple options, the solution to the decision process is considered to come to mind by way of logic and exhaustively sensible considerations of probable consequences. Thus, System 2 processing is here thought to not need or even to not involve any affective contribution.

Despite the large number of contributions that support the dual-systems view both theoretically and empirically, such theories have nevertheless recently come under strong fire (Keren and Schul, 2009; Kruglanski and Gigerenzer, 2011). The main point of criticism put forward by Keren and Schul (2009, p. 534) is that “the different dual-system theories lack conceptual clarity, that they are based upon methodological methods that are questionable, and that they rely on insufficient (and often inadequate) empirical evidence.” Kruglanski and Gigerenzer (2011) provide a unified approach and explain that both, intuition and deliberation, rely on the same functional principles (i.e., they are based on if – then rules), which is dependent on environmental conditions. As a reply to such criticism, Evans and Stanovich (2013) recently riposted that it is overstated since such criticism refers to dual-system models as a class of purely the same theoretical assumptions. They clarify that there are indeed different assumptions and terminologies subsumed under the dual-system framework, which needs to be considered. Nevertheless, there is also neuronal evidence against the assumptions of the dual-system approach (Mega et al., 2015). The authors did a functional-magnetic-resonance-imaging study and asked participants to judge either intuitively or deliberately the authenticity of emotional facial expressions. Interestingly, the authors found that intuition and deliberation recruit the same neuronal networks – a finding well in line with Kruglanski and Gigerenzer’s (2011) proposal. It can be summarized that the dual-system framework is being much debated at the moment (see also volume 8 of *Perspectives on Psychological Science*, 2013) and therefore, it is very likely that there will be a revised conception in the foreseeable future.

### Dual-System Models and the Discontinuity Model of Insight: Intuition As the First and Biased Problem Representation

After having shortly named the key assumptions of the dual-system framework as well as potential critical points, we will continue by elaborating on why we think the experimental approach of the insight problem solving literature (e.g., Danek et al., 2013) is similar to the one pursued by the heuristics-and-biases framework (Kahneman, 2011). A typical task used by researchers of the heuristics-and-biases approach is *the bat and the ball problem*. Participants are told that a bat and ball together cost $ 1.10 in total and that the bat costs $ 1 more than the ball. Then they are asked to state how much the ball costs. A vast number of experiments showed that the first “intuitive answer,” following Kahneman’s terminology, is 10 cent, but after a while of conscious deliberation (i.e., analytical thought) participants find out that the correct answer is 5 cent (Kahneman, 2011). Here is employed the same principle as in the magic-trick paradigm: the first and rapidly formed judgment, which is intentionally induced by the task material, is incorrect and hampers the generation of the correct solution (here 5 cent). In terms of the representational change theory an over-constraint problem representation is activated, where a simple goal representation is set up: total sum minus bat results immediately in the cost of the ball. Overcoming these assumptions seems difficult and requires a more sophisticated goal representation that combines two sets of information: (1) bat - ball = 1 AND (2) bat + ball = 1.10 => 1 in (2) ball + ball + 1 = 1.10 => ball = 0.05).

Together, experiments from both scientific fields show that by exploiting peoples’ intuitive apprehension of a problem, the solution is precluded from the beginning. To overcome the impasse or bias, it is suggested that the problem solver may engage in restructuring the problem space or in analytic strategies so as to eventually being able to solve the problem and to arrive at the objectively correct answer. Thus, there might be a reasonable mapping of the discontinuity model to the common dual-system model: first, the intuitive system starts (whether by default first or in parallel to System 2), and will lead to an over-constrained or biased problem representation that subsequently may lead to an impasse or conflict. Essential for reaching a solution is, (i) that the problem solver or decision maker realizes that the fast initial apprehension of the problem precludes its solution and (ii) engages in a representational change to overcome the initial problem representation (Öllinger et al., 2014). Since, by definition, System 2 processing is slower than System 1 processing it can smooth out the first and hasty attempts made by System 1. In the diction of dual system theorists, the analytic mind is called up when encountering an impasse or conflict and will attempt to deliberately solve the problem by applying certain rational strategies. Importantly, Systems 1 and 2, or intuition and insight, are here considered to be qualitatively different – “hare and tortoise.”

Equally important, System 1 is considered subordinate to System 2 and its hasty responses needs to be tamed (cf. Kahneman, 2011, p. 185). Kahneman (2011, p. 44) states: “One of the main functions of System 2 is to monitor and control thought and actions “suggested” by System 1, allowing some to be expressed directly in behavior and suppressing or modifying others.” Given such an understanding of intuition and insight, the discontinuity model may suffer from the very same conceptual problem as a dual-system account of reasoning: that is, how and by which factors is a conflict or impasse detected? “Who” eventually launches restructuring processes that are needed to overcome the error? How does restructuring of the first problem representation take place? This may be viewed as a variation of the “homunculus problem.”

Hence, within the discontinuity conception of insight, intuition is not regarded as helpful or diagnostic for the generation of a pending insight. In line with this idea, Metcalfe and Wiebe (1987) investigated feeling of warmth accompanying insight and incremental problem solving using classical insight problems and algebraic problems. They used feeling-of-warmth ratings as the assessment of how close participants intuitively felt to the solution, which was taken to indicate the *subjective nearness to the solution*. Interestingly, they found out that these subjective feelings of warmth differed for insight and non-insight solutions insofar that they could predict performance only on incremental algebra problems. For insight problems such intuitive feelings were lacking. Given this result, one may conclude that intuition differs from insight concerning the (introspective) access to non-conscious processing: whereas decision makers intuit the solution to a problem, people solving the problem by insight show to lack such hunches. Thus, additionally to the continuity/discontinuity distinction, insightful solutions as in contrast to intuitive ones seem to be discrete phenomena in terms of availability to awareness. However, it could be also possible that the conscious assessment of how close/far the solution is, just easier for non-insight tasks. Since non-insight tasks are well-defined insofar that there are clear starts, solution paths, and goals, which enables exact planning of the necessary steps and its order (as for example in algebraic problems). Conversely, classical insight problems may be technically well-defined (in that there is also a clear start and goal, see e.g., the famous nine-dot problem), but since the problem’s different components are unhelpfully represented in the problem solvers mental set, it is difficult or rather impossible to estimate how far/close the solution is.

### Interim Summary III

As an interim summary, it may be concluded that intuition research advocates a continuity model, in which intuition and insight build upon each other in a gradual and cumulative fashion: people are non-consciously sensitized toward pattern or meaning in the environment and act accordingly (e.g., Bowers et al., 1990). In contrast, insight research focuses on a discontinuity model, in which the initial representation of the problem (i.e., early intuition) biases later solution attempts and has to be overcome in order to reach a solution. Here, no intuitive precursors of insight in terms of a subjectively felt nearness toward the solution are assumed. This latter model resembles famous, yet recently heavily criticized, dual-system models in judgment and decision-making research insofar as in both approaches the participants first intuitive apprehension of a problem biases its later solution.

## Semantic Coherence Tasks Used in Intuition and Insight Research: Word Triads and Remote Associates

Interestingly, in the semantic domain, intuition research following Bowers et al. (1990) approach *and* contemporary insight research do have used similar stimuli yet with different task rationales, which could be used as an excellent starting point for necessary, and up to now lacking, common investigations. As described earlier in this contribution, in the tradition of Bowers et al. (1990, 1995), typical coherence judgment tasks include semantically coherent and incoherent word triads – a task that dates back to the work of Mednick (1962). Here, response patterns of both triad types (i.e., coherent vs. incoherent) are compared to each other. In recent research on insight problem solving, Bowden et al. (2005) presented a novel framework and a new class of problems in order to probe insight problem solving. The authors equate subjectively reported aha-experiences with insight. The authors have used word triads based on Mednick’s (1962) task to investigate the neuronal underpinnings of insight. They presented a large number of problems that can be solved either by insight or by non-insight (i.e., Aha! vs. Non-Aha!) and do not require a lot of time to be solved (Kounios and Beeman, 2014). As a result they found that Aha! solutions revealed distinguish neural patterns than Non-Aha!-solutions. Unlike intuition research, they (1) only applied word triads that are principally solvable (i.e., no incoherent triads), and (2) word triads that consist of compound remote associate.

Bowers et al. (1990), distinguished two types of triads and termed them *convergent and divergent triads*, respectively. For convergent triads the common associate means the same with respect to each clue word, whereas for divergent triads the common associate is more remote and changes its meaning with respect to each clue word. An example for a coherent convergent triad is SALT DEEP FOAM– SEA; and an example for a divergent triad is AGE MILE SAND– STONE. Unlike convergent triads, divergent triads are built in a way one need to detect the multiple meanings of the solution word to associate it with the meanings of the three clue words. As divergent triads may require a restructuring of the different meanings of the clues with respect to the solution, these kinds of triads could be nicely seen as an insight condition.

According to Bowden and Jung-Beeman (2007), divergent triads are not as complex as classical insight problems, but they can nevertheless be used as a kind of insight problems. Like typical insight tasks (1) they misdirect retrieval processes (i.e., the first word of a divergent triad biases later thought toward a specific, yet wrong direction), (2) the strategy that has led to the correct solution cannot be reported by the problem solver, and (3) aha-experiences can occur.

For such divergent triads, Cranford and Moss (2012), using a verbal protocol method, found out that there are two different types of insight problems, for which only one type shows the typical traditional characteristics of an insight. It has to be emphasized that, unlike Bowden et al. (2005), the authors consider all three components, subjective aha-experience, impasse, and restructuring, as necessary for an insight to occur. They could show that some problems, consisting of divergent triads, could be solved via *immediate insight*, whereas others were solved by non-immediate or *delayed insight*. Interestingly, only the latter type of insights showed the supposed phases of insight. Fedor et al. (2015) detailed on this question and found that the classical insight sequence (i.e., constrained search, impasse, insight, extended search, and solution) is a rather rare event. They found that participants showed much more often fairly different insight sequences (i.e., a flexible order of the different problem-solving stages), which has to be further specified in the future. We consider this line of research (Cranford and Moss, 2012; Kounios and Beeman, 2014; Fedor et al., 2015) as promising and important for future endeavors, which may initiate the common investigations of intuition and insight.

## Conclusion, Open Research Questions, and Future Directions

To conclude, we set out to disentangle the underlying mechanisms of intuition and insight so as to clarify their relationship. At first sight, intuition and insight seem to be very differently conceptualized: while the intuition literature favors a continuity model, insight has been described within in a discontinuity model. In a continuity model, early (semantic) readout processes are taken as diagnostic for the non-conscious detection of environmental patterns and/or meaning (in terms of an antecedent of later explicit mental representation or insight). Intuition is described as aiding decision making and problem solving when time and cognitive capacity is limited and necessary information is temporarily unavailable. Contrary to this, in a discontinuity model early intuitive responses misdirect the generation of a correct solution or are experimentally utilized to bias solution attempts. In this case, intuitions lead people astray. Instead of employing intuition, mental restructuring processes (i.e., qualitative changes in the non-conscious search processes) are needed to overcome biased intuitive impressions or apprehensions so as to eventually solve the problem. In that respect, a discontinuity model resembles dual-process accounts in judgment and decision making.

Except early work by Bowers et al. (1990, 1995) and Dorfman et al. (1996), there have not been much empirical investigations so far aiming at exploring similarities and differences in the underlying neurocognitive mechanisms of intuition and insight. A major drawback here may be that there are no tasks that easily enable a direct empirical comparison between the two concepts. Nevertheless, we consider it very important to test intuitive and insight solution processes by means of exactly the same task and within the same participants. Such a task needs to be created. With this theoretical contribution, we therefore aim to initiate common investigations of both fields of research to detect neurocognitive similarities and differences between intuitive processing and insight problem solving. A good starting point for common empirical investigations may be the use of different types of triads [as for example divergent and convergent triads, as formerly suggested by Bowers et al. (1990)] in order to induce gradual and discontinuous solution attempts. We also consider it important to investigate not only the cognitive processes that may underlie intuition and insight, but also the neuronal processes involved. Future studies may shed light on the specific (and maybe distinct) neuronal correlates, which will then also allow drawing conclusions about the theoretical conceptualization of the two phenomena. Interesting research questions would be (as non-exhaustive list): (1) Are the neuronal correlates different for the two types of triads (convergent versus divergent triads)? (2) Do aha-experiences also occur for convergent triads? (3) Do feelings-of-warmth ratings occur for both types of triads or only for convergent triads? (4) Do verbal protocols differ for the two types of triads? (5) How can the assumed recursive coherence building process be neuronally mapped? The further investigation of the underlying cognitive and neuronal processes of restructuring may also deeply progress our understanding of the topic. Here, Öllinger et al. (2006, 2013) reached influential results that may be carried forward in future research. Equally important, following Kounios and Beeman (2014) in using current neuroimaging techniques may promote the detection of objective physiological markers of insight (in form of a specific neuronal or electrophysiological activation pattern accompanying the experience of impasses and aha’s as well as correlating mental restructuring processes). Kounios and Beeman (2014) as well as Sandkühler and Bhattacharya (2008) already gained promising results in this respect, thus their research may be a good starting point for the future. To sum up, intuition and insight are intriguing (non-analytical) mental phenomena that need to be further investigated in the future.

## Author Contributions

TZ developed the theoretical conception; wrote the article. MÖ developed the theoretical conception; revised the manuscript. KV developed the theoretical conception; revised the manuscript.

## Conflict of Interest Statement

The authors declare that the research was conducted in the absence of any commercial or financial relationships that could be construed as a potential conflict of interest.

## Footnotes

1 There is the idea that a period, in which a person after encountering an impasse is not being consciously engaged in finding the solution anymore and puts the problem aside (i.e., the *incubation period*) fosters sudden insights of the solution (e.g., Gilhooly et al., 2012). Ritter and Dijksterhuis (2014) explain that unconscious thought processes continue to find the problem’s solution by re-organizing memory content eventually resulting in gist-based representations. This occurs in the absence of a person’s conscious attempts. It has to be emphasized, however, that empirical studies revealed different results as to whether incubation periods are beneficial for problem solving. The specific conditions under which positive incubation effects take place have to be further investigated (Sio and Ormerod, 2009).

2 For the sake of completeness, it has to be emphasized that metacognitive processes may play a role as well in intuitive processing. To strengthen the scope of our argumentation, we decided not to detail on this notion. Please see Mealor and Dienes (2013); Storm and Hickman (2015), or Thompson et al. (2011). A particular emphasize may be laid on the concept of experience-based metacognitive feelings (e.g., Koriat and Levy-Sadot, 1999).

## References

[B1] Betsch T. (2008). “The nature of intuition and its neglect in research on judgment and decision making,” in *Intuition in Judgment and Decision Making*, eds Plessner H. Betsch C. Betsch T. (New York, NY: Lawrence Erlbaum Associates), 3–22.

[B2] Bolte A. Goschke T. (2005). On the speed of intuition: intuitive judgments of semantic coherence under different response deadlines. *Mem. Cognit.* 33 1248–1255. 10.3758/BF03193226 16532857

[B3] Bowden E. M. Jung-Beeman M. (2007). Methods for investigating the neural components of insight. *Methods* 42 87–99. 10.1016/j.ymeth.2006.11.007 17434419

[B4] Bowden E. M. Jung-Beeman M. Fleck J. Kounios J. (2005). New approaches to demystifying insight. *Trends Cogn. Sci.* 9 322–328. 10.1016/j.tics.2005.05.012 15953756

[B5] Bowers K. S. (1984). “On being unconsciously influenced and informed,” in *The Unconscious Reconsidered*, eds Bowers K. S. Meichenbaum D. (New York, NY: John Wiley & Sons), 227–272.

[B6] Bowers K. S. Farvolden P. Mermigis L. (1995). “Intuitive antecedents of insight,” in *The Creative Cognition Approach*, eds Smith S. M. Ward T. B. Finke R. A. (Cambridge, MA: The MIT Press), 27–51.

[B7] Bowers K. S. Regehr G. Balthazard C. Parker K. (1990). Intuition in the context of discovery. *Cognit. Psychol.* 22 72–110. 10.1016/0010-0285(90)90004-N

[B8] Claxton G. (1998). Investigating human intuition: knowing without knowing why. *Psychologist* 11 217–220. 10.3758/s13415-014-0286-7

[B9] Collins A. M. Loftus E. F. (1975). A spreading-activation theory of semantic processing. *Psychol. Rev.* 82 407–428. 10.1037/0033-295X.82.6.407

[B10] Cranford E. A. Moss J. (2012). Is insight always the same? A verbal protocol analysis of insight in compound remote associate problems. *J. Probl. Solving* 4 128–153.

[B11] Danek A. H. Fraps T. von Müller A. Grothe B. Öllinger M. (2013). Aha! experiences leave a mark: facilitated recall of insight solutions. *Psychol. Res.* 77 659–669. 10.1007/s00426-012-0454-8 23007629

[B12] Danek A. H. Wiley J. Öllinger M. (2016). Solving classical insight problems without Aha! experience: 9 dot, 8 coin, and matchstick arithmetic problems. *J. Probl. Solving* 9 47–57. 10.7771/1932-6246.1183

[B13] Dorfman J. Shames V. A. Kihlstrom J. F. (1996). “Intuition, incubation and insight: implicit cognition in problem solving,” in *Implicit Cognition*, ed. Underwood G. (Oxford: Oxford University Press), 257–296.

[B14] Duncker K. (1935). *Zur Psychologie des Produktiven Denkens [On the psychology of productive Thinking].* Berlin: Springer.

[B15] Duncker K. (1945). On problem solving. *Psychol. Monogr.* 58:270 10.1037/h0093599

[B16] Durso F. T. F. Rea C. C. B. Dayton T. (1994). Graph-theoretic confirmation of restructuring during insight. *Psychol. Sci.* 5 94–97. 10.1111/j.1467-9280.1994.tb00637.x

[B17] Evans J. Frankish K. (eds) (2009). *In Two Minds: Dual Processes and Beyond*. Oxford: Oxford University Press.

[B18] Evans J. Stanovich K. S. (2013). Dual-process theories of higher cognition: advancing the debate. *Perspect. Psychol. Sci.* 8 223–241. 10.1177/1745691612460685 26172965

[B19] Fedor A. Szatmary E. Öllinger M. (2015). Problem solving stages in the five square problem. *Front. Psychol.* 6:1050 10.3389/fpsyg.2015.01050 PMC4523725 26300794

[B20] Gick M. L. Lockhardt R. S. (1995). “Cognitive and affective components of insight,” in *The Nature of Insight*, eds Sternberg R. J. Davidson J. E. (Cambridge, MA: The MIT Press), 197–228.

[B21] Gigerenzer G. (2008). *Gut Feelings: The Intelligence of the Unconscious*. New York, NY: Viking.

[B22] Gigerenzer G. Gaissmaier W. (2011). Heuristic decision making. *Annu. Rev. Psychol.* 62 451–482. 10.1146/annurev-psych-120709-145346 21126183

[B23] Gigerenzer G. Todd P. M. The Abc Research Group (eds) (1999). *Simple Heuristics that Make us Smart.* New York, NY: Oxford University Press.

[B24] Gilhooly K. J. Georgiou G. J. Garrison J. Reston J. D. Sirota M. (2012). Don’t wait to incubate: immediate versus delayed incubation in divergent thinking. *Mem. Cognit.* 40 966–975. 10.3758/s13421-012-0199-z 22382649

[B25] Gladwell M. (2005). *Blink. The Power of Thinking Without Thinking.* London: Penguin Books.

[B26] Glöckner A. Witteman C. (2010). “Foundations for tracing intuition: models, findings, categorizations,” in *Foundations for Tracing Intuition: Challenges and Methods*, eds Glöckner A. Witteman C. (East Sussex: Psychology Press), 1–23.

[B27] Hicks J. A. Burton C. M. Cicero D. C. Trent J. King L. A. (2010). Positive affect, intuition, and feelings of meaning. *J. Pers. Soc. Psychol.* 89 967–979. 10.1037/a0019377 20515252

[B28] Hogarth R. M. (2001). *Educating Intuition.* Chicago, IL: University of Chicago Press.

[B29] Jung-Beeman M. Bowden E. M. Haberman J. Frymiare J. L. Arambel-Liu S. Greenblatt R. (2004). Neural activity when people solve verbal problems with insight. *PLoS Biol.* 2:E97 10.1371/journal.pbio.0020097 PMC387268 15094802

[B30] Kahneman D. (2011). *Thinking, Fast and Slow*. London: Penguin Books.

[B31] Kahneman D. Tversky A. (1974). Judgment under uncertainty: heuristics and biases. *Science* 185 1124–1131. 10.1126/science.185.4157.1124 17835457

[B32] Keren G. Schul Y. (2009). Two is not always better than one: a critical evaluation of two-system theories. *Perspect. Psychol. Sci.* 4 533–550. 10.1111/j.1745-6924.2009.01164.x 26161732

[B33] Kershaw T. C. Ohlsson S. (2004). Multiple causes of difficulty in insight: the case of thenine-dot problem. *J. Exp. Psychol. Learn. Mem. Cogn.* 30 3–13. 10.1037/0278-7393.30.1.3 14736292

[B34] Kizilirmak J. Galvao Gomes da Silva J. Imamoglu F. Richardsohn-Klavehn R. (2015). Generation and the subjective feeling of “aha!” are independently related to learning from insight. *Psychol. Res.* 10.1007/s00426-015-0697-2 [Epub ahead of print]. PMC5069302 26280758

[B35] Klein G. Jarosz A. (2011). A naturalistic study of insight. *J. Cogn. Eng. Decis. Mak.* 5 335–351. 10.1177/1555343411427013

[B36] Knoblich G. Ohlsson S. Haider H. Rhenius D. (1999). Constraint relaxation and chunk decomposition in insight problem solving. *J. Exp. Psychol. Learn. Mem. Cogn.* 25 1534–1555. 10.1037/0278-7393.25.6.1534

[B37] Knoblich G. Ohlsson S. Raney G. E. (2001). An eye movement study of insight problem solving. *Mem. Cognit.* 29 1000–1009. 10.3758/BF03195762 11820744

[B38] Knoblich G. Öllinger M. (2006). “Einsicht und umstrukturierung beim problemlösen [insight and restructering in problem solving],” in *Denken und Problemlösen. Enzyklopädie der Psychologie [Thinking and Problem Solving. Encyclopedia of Psychology]*, ed. Funke J. (Göttingen: Hogrefe), 3–86.

[B39] Köhler W. (1921). *Intelligenzprüfungen am Menschenaffen [Investigating intelligence in great apes]*. Berlin: Springer.

[B40] Koriat A. Levy-Sadot R. (1999). “Processes underlying metacognitive judgments: information-based and experience-based monitoring of one’s own knowledge,” in *Dual-Process Theories in Social Psychology*, eds Chaiken S. Trope Y. (New York, NY: Guilford Press), 483–502.

[B41] Kounios J. Beeman M. (2014). The cognitive neuroscience of insight. *Annu. Rev. Psychol.* 65 71–93. 10.1146/annurev-psych-010213-115154 24405359

[B42] Kruglanski A. W. Gigerenzer G. (2011). Intuitive and deliberate judgments are based on common principles. *Psychol. Rev.* 118 97–109. 10.1037/a0020762 21244188

[B43] Lieberman M. D. (2000). Intuition: a social cognitive neuroscience approach. *Psychol. Bull.* 126 109–137. 10.1037/0033-2909.126.1.109 10668352

[B44] Luchins A. S. Luchins E. H. (1959). *Rigidity of Behavior: A Variational Approach to the Effect of Einstellung*. Eugene, OR: University of Oregon Books.

[B45] Maier N. R. F. (1930). Reasoning in humans. I. On direction. *J. Comp. Psychol.* 10 115–143. 10.1037/h0073232

[B46] Mangan B. (1993). Taking phenomenology seriously: the “fringe” and its implications for cognitive research. *Consci. Cogn.* 2 89–108. 10.1006/ccog.1993.1008

[B47] Mangan B. (2001). Sensation’s ghost. The non-sensory “fringe” of consciousness. *Psyche* 7.

[B48] Mangan B. (2015). The uncanny valley as fringe experience. *Interact. Stud.* 16 193–199. 10.1075/is.16.2.05man

[B49] Mealor A. D. Dienes Z. (2013). The speed of metacognition: taking time to get to know one’s structural knowledge. *Conscious. Cogn.* 22 123–136. 10.1016/j.concog.2012.11.009 23262257

[B50] Mednick S. A. (1962). The associative basis of the creative process. *Psychol. Rev.* 69 220–232. 10.1037/h0048850 14472013

[B51] Mega L. F. Gigerenzer G. Volz K. G. (2015). Do intuitive and deliberate judgments rely on two distinct neural systems? A case study in face processing. *Front. Hum. Neurosci.* 9:456 10.3389/fnhum.2015.00465 PMC4548224 26379523

[B52] Metcalfe J. Wiebe D. (1987). Intuition in insight and noninsight problem solving. *Mem. Cognit.* 15 238–246. 10.3758/BF03197722 3600264

[B53] Metzger W. (1953). *Gesetze des Sehens [Rules of vision].* Frankfurt: Kramer.

[B54] Newell A. Simon H. A. (1972). *Human Problem Solving.* Englewood Cliffs, NJ: Prentice Hall.

[B55] Norman E. (2002). Subcategories of “fringe consciousness” and their related nonconscious contexts. *Psyche* 8 1–15.

[B56] Norman E. (2016). Metacognition and minfulness: the role of fringe consciousness. *Mindfulness* 10.1007/s12671-016-0494-z PMC5241337 28163796

[B57] Norman E. Price M. C. Duff S. C. (2006). Fringe consciousness in sequence learning: the influence of individual differences. *Consci. Cogn.* 15 723–760. 10.1016/j.concog.2005.06.003 16154763

[B58] Norman E. Price M. C. Duff S. C. (2010). “Fringe consciousness: a useful framework for clarifying the nature of experience-based feelings,” in *Trends and Prospects in Metacognition Research*, eds Efklides A. Misailidi P. (New York, NY: Springer), 63–89.

[B59] Ohlsson S. (1992). “Information-processing explanations of insight and related phenomena,” in *Advances in the Psychology of Thinking*, eds Keane M. Gilhooly K. (London: Harvester-Wheatsheaf), 1–44.

[B60] Ohlsson S. (2011). *Deep Learning. How the Mind Overrides Experience.* New York, NY: Cambridge University Press.

[B61] Öllinger M. Jones G. Faber A. H. Knoblich G. (2013). Cognitive mechanisms of insight: the role of heuristics and the representational change in solving the eight-coin problem. *J. Exp. Psychol. Learn. Mem. Cogn.* 39 931–939. 10.1037/a0029194 22799283

[B62] Öllinger M. Jones G. Knoblich G. (2006). Heuristics and representational change in two-move matchstick arithmetic task. *Adv. Cogn. Psychol.* 2 239–253. 10.2478/v10053-008-0059-3

[B63] Öllinger M. Jones G. Knoblich G. (2008). Investigating the effect of mental set on insight problem solving. *Exp. Psychol.* 55 270–282. 10.1027/1618-3169.55.4.269 18683624

[B64] Öllinger M. Jones G. Knoblich G. (2014). The dynamics of search, impasse, and representational change provide a coherent explanation of difficulty in the nine-dot problem. *Psychol. Res.* 78 266–275. 10.1007/s00426-013-0494-8 23708954

[B65] Ormerod T. C. MacGregor J. N. Chronicle E. P. (2002). Dynamics and constraints in insight problem solving. *J. Exp. Psychol. Learn. Mem. Cogn.* 28 791–799. 10.1037/0278-7393.28.4.791 12109769

[B66] Plessner H. Betsch C. Betsch T. (eds) (2008). *Intuition in Judgment and Decision Making.* New York, NY: Lawrence Erlbaum Associates.

[B67] Polanyi M. (1966). *Implizites Wissen [The tacit dimension].* Frankfurt: Suhrkamp.

[B68] Price M. C. (2002). Measuring the fringe of experience. *Psyche* 8 1–24.

[B69] Price M. C. Norman E. (2008). Intuitive feelings on the fringe of consciousness: are they conscious and does it matter? *Judgm. Decis. Mak.* 3 28–41.

[B70] Reber R. Ruch-Monachon M.-A. Perrig W. J. (2007). Decomposing intuitive components in a conceptual problem solving task. *Conscious. Cogn.* 16 294–309. 10.1016/j.concog.2006.05.004 16815036

[B71] Reber R. Schwarz N. Winkielmann P. (2004). Processing fluency and aesthetic pleasure: is beauty in the perceiver’s processing experience? *Pers. Soc. Psychol. Rev.* 8 364–382. 10.1207/s15327957pspr0804_3 15582859

[B72] Remmers C. Topolinski T. Dietrich D. E. Michalak J. (2014). Impaired intuition in pateints with major depressive disorder. *Br. J. Clin. Psychol.* 54 200–213. 10.1111/bjc.12069 25307321

[B73] Ritter S. Dijksterhuis A. (2014). Creativity – the unconscious foundations of the incubation period. *Front. Hum. Neurosci.* 8:215 10.3389/fnhum.2014.00215 PMC3990058 24782742

[B74] Sadler-Smith E. (2008). *Inside Intuition.* Abingdon: Routledge.

[B75] Sandkühler S. Bhattacharya J. (2008). Deconstructing insight: EEG correlates of insightful problem solving. *PLoS ONE* 3:1459 10.1371/journal.pone.0001459 PMC2180197 18213368

[B76] Seifert C. M. Meyer D. E. Davidson N. Patalano A. L. Yaniv I. (1995). “Demystification of cognitive insight: opportunistic assimilation and the prepared-mind perspective,” in *The Nature of Insight*, eds Sternberg R. J. Davidson J. E. (Cambridge, MA: The MIT Press), 65–124.

[B77] Sio U. N. Ormerod T. C. (2009). Does incubation enhance problem solving? A meta- analytic review. *Psychol. Bull.* 135 94–120. 10.1037/a0014212 19210055

[B78] Stanovich K. E. West R. F. (2000). Individual differences in reasoning: implications for the rationality debate. *Behav. Brain Sci.* 23 645–665. 10.1017/S0140525X00003435 11301544

[B79] Sternberg R. J. Davidson J. E. (eds) (1995). *The Nature of Insight.* Cambridge, MA: The MIT Press.

[B80] Storm B. C. Hickman M. L. (2015). Mental fixation and metacognitive predictions of insight in creative problem solving. *Q. J. Exp. Psychol.* 68 802–813. 10.1080/17470218.2014.966730 25230691

[B81] Thompson V. A. Prowse Turner J. A. Pennycook G. (2011). Intuition, reason, and metacognition. *Cognit. Psychol.* 63 107–140. 10.1016/j.cogpsych.2011.06.001 21798215

[B82] Topolinski S. Reber R. (2010). Gaining insight into the “aha” experience. *Curr. Dir. Psychol. Sci.* 19 402–405. 10.3389/fpsyg.2014.01408

[B83] Topolinski S. Strack F. (2008). Where there’s a will – there’s no intuition. The unintentional basis of semantic coherence judgments. *J. Mem. Lang.* 58 1032–1048. 10.1016/j.jml.2008.01.002

[B84] Topolinski S. Strack F. (2009a). Scanning the “fringe” of consciousness: what is felt and what is not felt in intuitions about semantic coherence. *Consci. Cogn.* 18 608–618. 10.1016/j.concog.2008.06.002 18650104

[B85] Topolinski S. Strack F. (2009b). The analysis of intuition: processing fluency and affect in judgments of semantic coherence. *Cogn. Emot.* 23 1465–1503. 10.1080/02699930802420745

[B86] Volz K. G. von Cramon D. Y. (2006). What neuroscience can tell about intuitive processes in the context of perceptual discovery. *J. Cogn. Neurosci.* 18 1–11. 10.1162/jocn.2006.18.12.2077 17129192

[B87] Volz K. G. Zander T. (2014). Primed for intuition? *Neurosci. Decis. Mak.* 1 26–34. 10.2478/ndm-2014-0001

[B88] Wertheimer M. (1959). *Productive Thinking.* New York, NY: Harper.

[B89] Zander T. Horr N. K. Bolte A. Volz K. G. (2015). Intuition as a gradual process: investigating intuition-based and priming-based decisions with fMRI. *Brain Behav.* 6:e00420 10.1002/brb3.420 PMC4834943 27110441

[B90] Zsambok C. E. Klein G. (eds) (1997). *Naturalistic Decision Making.* New York, NY: Routlege.

